# Structure of human nucleosome containing the testis-specific histone variant TSH2B

**DOI:** 10.1107/S2053230X14004695

**Published:** 2014-03-25

**Authors:** Takashi Urahama, Naoki Horikoshi, Akihisa Osakabe, Hiroaki Tachiwana, Hitoshi Kurumizaka

**Affiliations:** a Laboratory of Structural Biology, Graduate School of Advanced Science and Engineering, Waseda University, 2-2 Wakamatsu-cho, Shinjuku-ku, Tokyo 162-8480, Japan

**Keywords:** TSH2B, nucleosome, chromatin, testis

## Abstract

The human histone H2B variant TSH2B is highly expressed in testis and may function in the chromatin transition during spermatogenesis. In the present study, the crystal structure of the human testis-specific nucleosome containing TSH2B was determined at 2.8 Å resolution. A local structural difference between TSH2B and canonical H2B in nucleosomes was detected around the TSH2B-specific amino-acid residue Ser85. The TSH2B Ser85 residue does not interact with H4 in the nucleosome, but in the canonical nucleosome the H2B Asn84 residue (corresponding to the TSH2B Ser85 residue) forms water-mediated hydrogen bonds with the H4 Arg78 residue. In contrast, the other TSH2B-specific amino-acid residues did not induce any significant local structural changes in the TSH2B nucleosome. These findings may provide important information for understanding how testis-specific histone variants form nucleosomes during spermatogenesis.

## Abstract 2

The crystal structure of human nucleosome containing the testis-specific TSH2B variant has been determined. The TSH2B Ser85 residue does not interact with H4 in the nucleosome, and induces a local structural difference between TSH2B and H2B in nucleosomes.

## 1. Introduction   

In eukaryotes, genomic DNA is complexed with histone proteins and forms chromatin. The basic repeating unit of chromatin is the nucleosome, in which the histone octamer, containing two each of the canonical histones H2A, H2B, H3 and H4, binds and wraps about 145–147 base pairs of DNA (Luger *et al.*, 1997 ▶). These canonical histones are produced during the S-phase of the cell cycle (Marzluff *et al.*, 2008 ▶).

In addition to the canonical histones, H2A, H2B and H3 variants with production that is not restricted to the S-phase have been identified (Talbert *et al.*, 2012 ▶). The mammalian histone H2B variant TSH2B was initially found in histone extracts from rat testes (Branson *et al.*, 1975 ▶; Shires *et al.*, 1975 ▶). Genomic and genetic analyses revealed that the mouse TSH2B homologue, TH2B, functions in genome-wide histone–protamine exchange during spermatogenesis (Govin *et al.*, 2007 ▶; Montellier *et al.*, 2013 ▶). In mice, TSH2B replaces the canonical H2B in early spermatogenesis and promotes a stepwise nucleosome-to-protamine transition (Montellier *et al.*, 2013 ▶). These facts suggested that TSH2B may form an unstable nucleosome for enhanced histone–protamine exchange. Consistently, previous biochemical experiments revealed that human TSH2B does not form a stable histone octamer, although the stabilities of the TSH2B and canonical H2B nucleosomes are similar (Li *et al.*, 2005 ▶).

To clarify the structure of the nucleosome containing TSH2B, we prepared the recombinant human TSH2B protein and reconstituted it into nucleosomes. We then determined the crystal structure of the TSH2B nucleosome at 2.8 Å resolution.

## 2. Materials and methods   

### 2.1. Purification of human histones TSH2B, H2A, H2B, H3.1 and H4   

**Figure 1:**
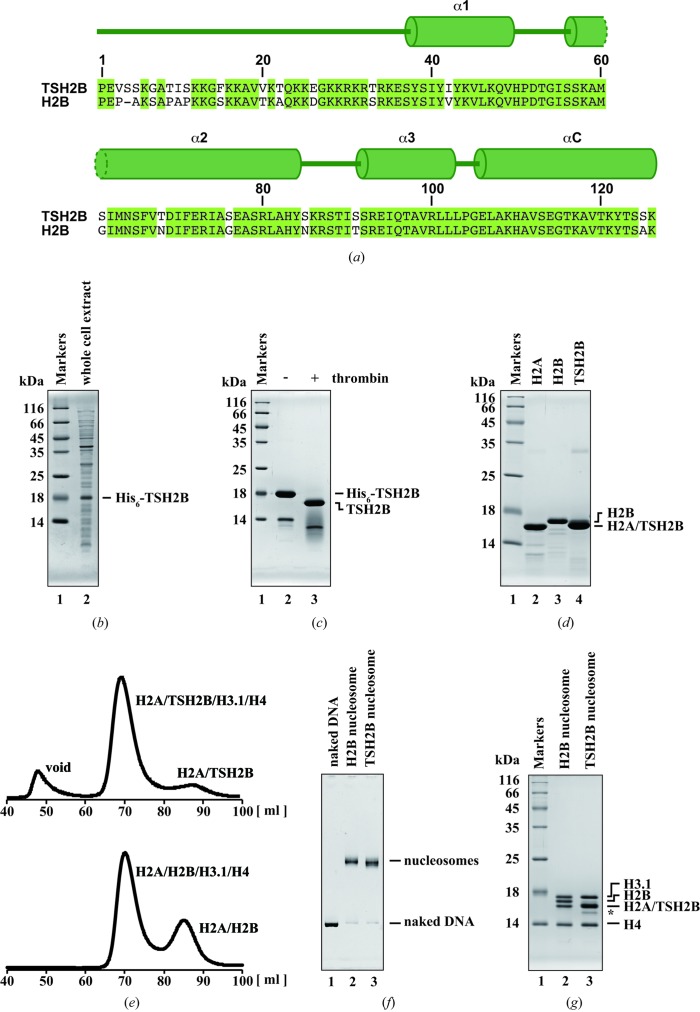
Reconstitution of the human testis-specific TSH2B nucleosome. (*a*) Alignment of the human H2B and TSH2B amino-acid sequences. The secondary structure of TSH2B in the nucleosome is shown at the top of the panel. Amino-acid residues that differ between H2B and TSH2B are represented with a white background. (*b*) Expression of His_6_-tagged TSH2B. Lane 1 contains molecular-mass markers. Lane 2 contains the whole-cell extract of the *E. coli* cells producing His_6_-tagged TSH2B. The proteins were analyzed by 16% SDS–PAGE with Coomassie Brilliant Blue staining. (*c*) The Ni–NTA agarose column fraction. Lane 1 contains molecular-mass markers. His_6_-tagged TSH2B eluted from the Ni–NTA agarose column was analyzed by 16% SDS–PAGE with Coomassie Brilliant Blue staining (lane 2). The TSH2B sample after His_6_-tag removal by thrombin protease was analyzed by 16% SDS–PAGE with Coomassie Brilliant Blue staining (lane 3). (*d*) The purified H2A (lane 2), H2B (lane 3) and TSH2B (lane 4) were analyzed by 18% SDS–PAGE with Coomassie Brilliant Blue staining. Lane 1 contains molecular-mass markers. (*e*) Analysis of the histone octamers by gel-filtration chromatography. H2A, H3.1, H4 and TSH2B or H2B were incubated without DNA in the presence of 2 *M* NaCl. The samples were then subjected to HiLoad 16/60 Superdex 200 gel-filtration column chromatography. The gel-filtration profile of the histone octamer reconstituted with H2A, TSH2B, H3.1 and H4 is shown in the upper panel, and that of the histone octamer reconstituted with H2A, H2B, H3.1 and H4 is shown in the lower panel. (*f*) Purified canonical H2B (lane 2) and TSH2B (lane 3) nucleosomes were analyzed by 6% nondenaturing polyacrylamide gel electrophoresis (PAGE). DNA was visualized by ethidium bromide staining. Lane 1 indicates naked DNA. (*g*) The histone compositions of the purified H2B (lane 2) and TSH2B (lanes 3) nucleosomes were analyzed by 18% SDS–PAGE. Histones were visualized by Coomassie Brilliant Blue staining. A trace amount of degradation products was incorporated into the TSH2B nucleosome, as indicated by an asterisk. Lane 1 contains molecular-mass markers.

The DNA fragment encoding human histone TSH2B was inserted into the pUC19 derivative vector previously used for the expression of canonical H2B (Tanaka *et al.*, 2004 ▶; Tachiwana *et al.*, 2011 ▶). *Escherichia coli* BL21(DE3) cells freshly transformed with the vector encoding TSH2B were grown on LB plates containing ampicillin (100 µg ml^−1^) at 310 K. Between five and 20 colonies grown on the LB plates were collected and inoculated into LB medium (2 l) containing ampicillin (50 µg ml^−1^). The *E. coli* cells expressing recombinant TSH2B as a hexahistidine (His_6_)-tagged protein were collected and resuspended in 50 ml 50 m*M* Tris–HCl buffer pH 7.5, containing 500 m*M* NaCl, 1 m*M* PMSF, 5% glycerol (Fig. 1 ▶ *b*). The cell suspension was sonicated and then centrifuged at 39 191*g* for 15 min at 277 K. The supernatants were discarded and the pellets were resuspended in 50 ml 50 m*M* Tris–HCl buffer pH 7.5 containing 500 m*M* NaCl, 1 m*M* PMSF, 5% glycerol. The resuspended sample was sonicated again and the pellets were collected by centrifugation at 39 191*g* for 15 min at 277 K. The His_6_-tagged TSH2B protein was harvested as insoluble inclusion bodies. 

The pellets were resuspended in 50 ml 50 m*M* Tris–HCl buffer pH 7.5 containing 500 m*M* NaCl, 5% glycerol, 7 *M* guanidine hydrochloride and were dissolved by sonication. The resuspended pellets were stirred overnight and the supernatant was obtained by centrifugation at 39 191*g* for 15 min at 277 K. The supernatant including the His_6_-tagged TSH2B was mixed with 4 ml (50% slurry) nickel–nitrilotriacetic acid (Ni–NTA) agarose resin (Qiagen) and the sample was rotated for 1 h at 277 K. The beads were then packed into an Econo-column (Bio-Rad) and washed with 100 ml 50 m*M* Tris–HCl buffer pH 8.0 containing 500 m*M* NaCl, 5% glycerol, 6 *M* urea, 5 m*M* imidazole. The His_6_-tagged TSH2B was eluted with a 100 ml linear gradient of 5–500 m*M* imidazole and dialyzed against 10 m*M* Tris–HCl buffer pH 7.5 containing 2 m*M* β-mercaptoethanol. The purity of TSH2B was about 70% after Ni–NTA agarose column chromatography (Fig. 1 ▶ *c*, lane 2) and the protein was further purified by Mono S column chromatography (GE Healthcare). After dialysis, the His_6_ tag was removed by thrombin protease (2.7 unit per milligram of protein; GE Healthcare) at room temperature for 3 h (Fig. 1 ▶ *c*, lane 3) and TSH2B was subjected to Mono S column chromatography (GE Healthcare). The column was washed with 20 m*M* sodium acetate pH 5.2 containing 200 m*M* NaCl, 2 m*M* β-mercaptoethanol, 1 m*M* EDTA, 6 *M* urea. TSH2B was then eluted by a linear gradient of 200–800 m*M* NaCl. TSH2B was highly purified, although trace amounts of degradation products and a TSH2B dimer were detected (Fig. 1 ▶ *d*, lane 4). The purified histones were dialyzed against 2 m*M* β-mercaptoethanol and freeze-dried. According to this method, several milligrams of TSH2B were obtained from 2 l LB culture. The other human histones (H2A, H2B, H3.1 and H4) were purified as described previously (Tachiwana *et al.*, 2011 ▶).

### 2.2. Preparation of the testis-specific nucleosome containing TSH2B   

To reconstitute the histone octamer containing TSH2B, purified H2A, H3.1, H4 and TSH2B were mixed in 20 m*M* Tris–HCl buffer pH 8.0 containing 7 *M* guanidine hydrochloride, 20 m*M* β-mercapto­ethanol, and rotated at 277 K for 2 h. The sample was dialyzed against 10 m*M* Tris–HCl buffer pH 7.5 containing 1 m*M* EDTA, 2 *M* NaCl, 5 m*M* β-mercaptoethanol. The resulting histone octamers were fractionated by HiLoad 16/60 Superdex 200 gel-filtration column chromatography (GE Healthcare) in 10 m*M* Tris–HCl buffer pH 7.5 containing 1 m*M* EDTA, 2 *M* NaCl, 5 m*M* β-mercaptoethanol. The elution volumes of the histone octamer containing TSH2B and the canonical histone octamer were the same (Fig. 1 ▶ *e*). This suggested that the histone stoichiometries in the octamers containing TSH2B and the canonical histone octamers are equivalent. The purified histone octamer containing TSH2B was mixed with the 146 base-pair DNA. After the sample was dialyzed against 10 m*M* Tris–HCl buffer (pH 7.5) containing 1 m*M* EDTA, 2 *M* KCl, 1 m*M* DTT, and the KCl concentration was gradually decreased to 0.25 *M* during dialysis. The 146 base-pair DNA was derived from a palindromic human α-satellite DNA fragment (Luger *et al.*, 1997 ▶), which was previously used for structural studies of human nucleosomes containing canonical histone H2B (Tsunaka *et al.*, 2005 ▶; Tachiwana *et al.*, 2010 ▶, 2011 ▶). To remove the nonspecific histone–DNA aggregates, the samples were incubated at 328 K for 2 h. The TSH2B nucleosome was further purified by nondenaturing polyacrylamide gel electrophoresis using a Prep Cell apparatus (Bio-Rad) (Figs. 1 ▶ *f* and 1 ▶ *g*).

### 2.3. Crystallization and determination of the TSH2B nucleosome structure   

For crystallization, the purified nucleosomes were concentrated and dialyzed against 20 m*M* potassium cacodylate buffer pH 6.0 containing 1 m*M* EDTA. The crystals of the TSH2B nucleosome were grown by the hanging-drop method at 293 K. The hanging drop was formed by adding 1 µl nucleosome solution (at a concentration of 2.5–4.0 mg ml^−1^) to 1 µl crystallization solution (20 m*M* potassium cacodylate pH 6.0, 50 m*M* KCl, 75–155 m*M* MnCl_2_). The drops were equilibrated against 500 µl reservoir solution (20 m*M* potassium cacodylate pH 6.0, 35–40 m*M* KCl, 50–80 m*M* MnCl_2_). For data collection, the crystals were harvested in a reservoir solution containing 29% 2-methyl-2,4-pentanediol, 2% trehalose and were flash-cooled in a stream of nitrogen gas at 100 K. Data sets were collected on the SPring-8 BL41XU beamline (Harima, Japan). The data sets were processed and scaled using the *HKL*-2000 program suite (Otwinowski & Minor, 1997 ▶). The TSH2B nucleosome crystals belonged to the orthorhombic space group *P*2_1_2_1_2_1_ and contained one nucleosome per asymmetric unit. Unit-cell parameters are provided in Table 1 ▶.

The structure of the TSH2B nucleosome was solved to 2.8 Å resolution. The data were processed using the *CCP*4 program suite (Winn *et al.*, 2011 ▶). The structure of the TSH2B nucleosome was determined by molecular replacement with *Phaser* (McCoy *et al.*, 2007 ▶), using the coordinates of the human nucleosome structure (Tachiwana *et al.*, 2010 ▶; PDB entry 3afa) as the search model. All refinements were performed using *CNS* (Brünger *et al.*, 1998 ▶). After rigid-body refinement, the model was refined by iterative rounds of energy minimization and *B*-factor refinement, and by manual model building using *Coot* (Emsley & Cowtan, 2004 ▶). The Ramachandran plot of the final structure showed no outlying residues, as assessed with *RAMPAGE* (Lovell *et al.*, 2003 ▶). The data-collection and refinement statistics are summarized in Table 1 ▶. All structure figures were created using *PyMOL* (http://www.pymol.org). The atomic coordinates of the TSH2B nucleosome have been deposited in the RCSB Protein Data Bank as entry 3wkj.

## 3. Results and discussion   

### 3.1. TSH2B structure in the nucleosome   

**Figure 2:**
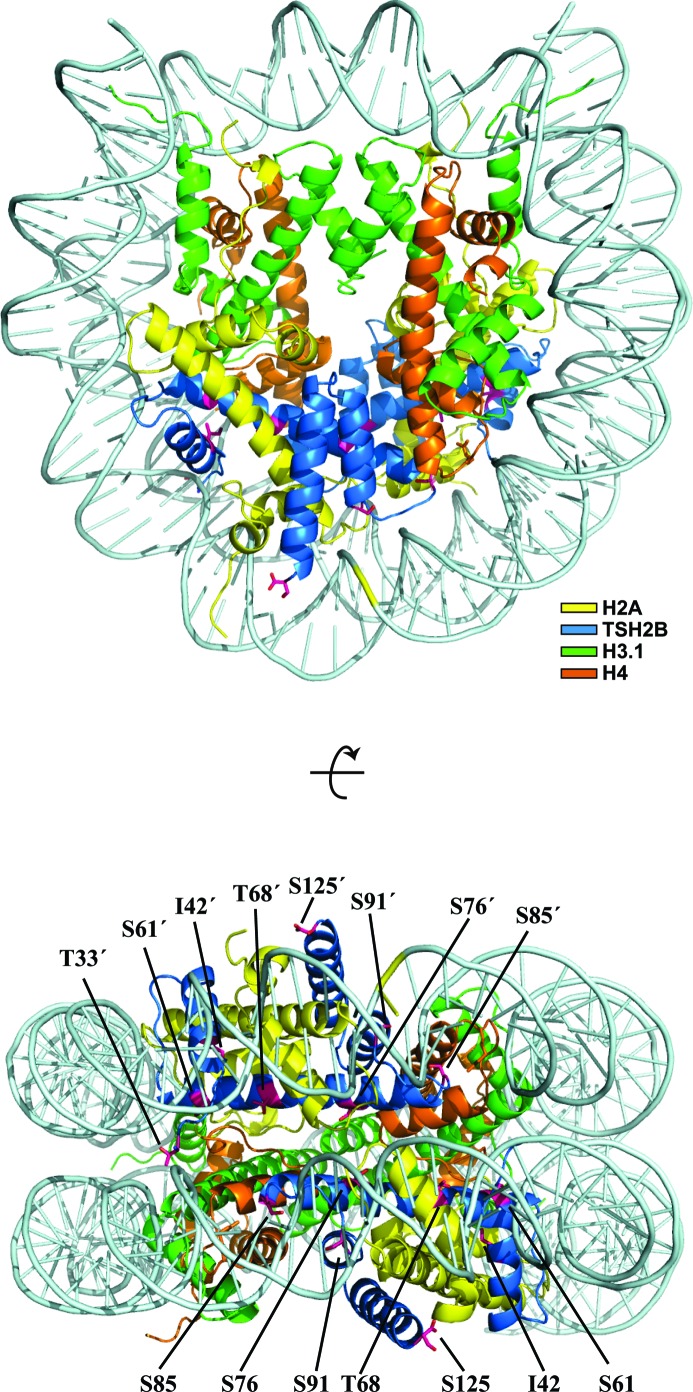
Crystal structure of the TSH2B nucleosome. Two views are presented, in which the TSH2B molecules are coloured blue and the H2A, H3.1 and H4 molecules are coloured yellow, green and orange, respectively. The TSH2B-specific residues are indicated. Dyad symmetry-related residues are denoted by a prime (′).

**Figure 3:**
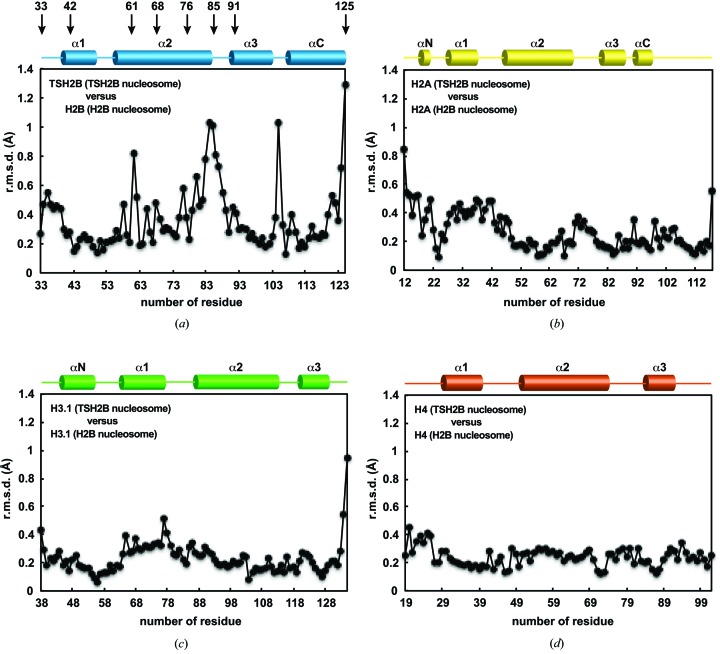
Structural differences between the histones in the TSH2B and canonical H2B nucleosomes. (*a*) The TSH2B and H2B structures were superimposed and the r.m.s.d. value for each residue pair was plotted. The secondary structure of TSH2B in the nucleosome is shown at the top of the panel. Arrows indicate the locations of the TSH2B-specific amino-acid residues. (*b*) The H2A structures of the TSH2B and H2B nucleosomes were superimposed and the r.m.s.d. value for each residue pair was plotted. The secondary structure of H2A in the nucleosome is shown at the top of the panel. (*c*) The H3.1 structures of the TSH2B and H2B nucleosomes were superimposed, and the r.m.s.d. value for each residue pair was plotted. The secondary structure of H3.1 in the nucleosome is shown at the top of the panel. (*d*) The H4 structures of the TSH2B and H2B nucleosomes were superimposed, and the r.m.s.d. value for each residue pair was plotted. The secondary structure of H4 in the nucleosome is shown at the top of the panel.

Human TSH2B contains 19 amino-acid differences compared with the canonical histone H2B (Fig. 1 ▶ *a*). We determined the crystal structure of the human testis-specific nucleosome containing TSH2B at 2.8 Å resolution (Fig. 2 ▶ and Table 1 ▶). TSH2B formed a dimer with H2A, and the two H2A–TSH2B dimers were symmetrically accommodated within the nucleosome (Fig. 2 ▶) as in the canonical nucleosome containing H2B. To compare the TSH2B and H2B structures in nucleosomes, the TSH2B structure was superimposed on the canonical H2B structure, and the r.m.s.d. for each residue pair was calculated and plotted (Fig. 3 ▶ *a*). A substantial structural difference was found around position 85 of TSH2B compared with the H2B structure. On the other hand, no substantial structural differences were observed in the H2A, H3.1 and H4 molecules between the TSH2B and canonical H2B nucleosomes (Figs. 3 ▶ *b*, 3 ▶ *c* and 3 ▶ *d*). Therefore, the local structural difference between TSH2B and H2B does not affect the H2A, H3.1 and H4 structures within the nucleosome.

### 3.2. Structural comparison of the regions with TSH2B-specific amino-acid residues between TSH2B and H2B nucleosomes   

**Figure 4:**
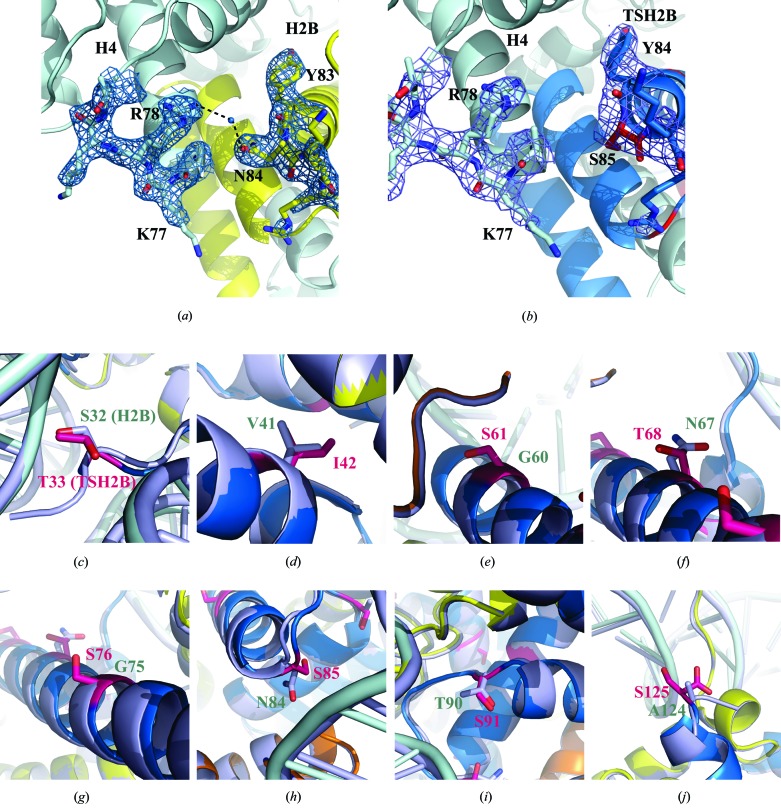
A close-up view of the structure around amino-acid residue Ser85 in TSH2B. (*a*) A close-up view around the canonical H2B Asn84 residue, which corresponds to the TSH2B Ser85 residue in the nucleosome (PDB entry 3afa; Tachiwana *et al.*, 2010 ▶). The H2B and H4 molecules are coloured yellow and pale cyan, respectively, and the side chains around the H2B Asn84 and H4 Arg78 residues are shown. The water-mediated hydrogen bonds between the H2B Asn84 and H4 Arg78 residues are depicted by dotted lines. The 2*mF*_o_ − *DF*_c_ maps of the regions around the H2B Asn84 and H4 Arg78 residues were calculated and contoured at the 1.5σ level. (*b*) A close-up view around the TSH2B Ser85 residue in the nucleosome. The TSH2B and H4 molecules are coloured blue and pale cyan, respectively, and the side chains around the TSH2B Ser85 and H4 Arg78 residues are shown. The 2*mF*_o_ − *DF*_c_ maps of the region around the TSH2B Ser85 and H4 Arg78 residues were calculated, and contoured at the 1.5σ level. (*c*)–(*j*) Close-up views of the TSH2B structure around the Thr33 (*c*), Ile42 (*d*), Ser61 (*e*), Thr68 (*f*), Ser76 (*g*), Ser85 (*h*), Ser91 (*i*) and Ser125 (*j*) residues. The corresponding regions of the H2B structure were superimposed. The TSH2B molecule is coloured blue, and the TSH2B Thr33, Ile42, Ser61, Thr68, Ser76, Ser85, Ser91 and Ser125 residues are coloured magenta. The H2B molecule is coloured grey.

A comparison of the TSH2B structure with the canonical H2B structure in nucleosomes revealed a local structural difference in TSH2B around the position of amino-acid residue 85 (Fig. 3 ▶ *a*). The Ser85 residue of TSH2B corresponds to the Asn84 residue of H2B. In the canonical nucleosome, the side chain of H2B Asn84 forms water-mediated hydrogen bonds to the side chain of H4 Arg78 (Fig. 4 ▶ *a*). This H2B–H4 interaction was not seen in the TSH2B nucleosome, because the H2B Asn84 residue was replaced with serine in TSH2B (Fig. 4 ▶ *b*). Although 19 amino-acid substitutions exist between TSH2B and H2B, 12 are in the unstructured N-terminal tail region (Fig. 1 ▶ *a*). The structures of the Thr33, Ile42, Ser61, Thr68, Ser76, Ser91 and Ser125 residues in TSH2B, which are located within the histonefold domain, were compared with those of the corresponding residues in H2B. Interestingly, no substantial structural differences in backbone geometry and side-chain orientation were observed around these seven substituted sites (residues Thr33, Ile42, Ser61, Thr68, Ser76, Ser91 and Ser125 in TSH2B), except around residue Ser85 (Figs. 4 ▶ *c*, 4 ▶ *d*, 4 ▶ *e*, 4 ▶ *f*, 4 ▶ *g*, 4 ▶ *h*, 4 ▶ *i* and 4 ▶ *j*). A previous biochemical study revealed that human TSH2B forms an unstable histone octamer with chicken H2A, H3 and H4 (Li *et al.*, 2005 ▶). The water-mediated hydrogen bonds between H2B Asn84 and H4 Arg78 in the canonical nucleosome are not formed in the TSH2B nucleosome. This local structural difference may affect the stability of the TSH2B nucleosome.

Most of the TSH2B-specific amino-acid residues exist in its unstructured N-terminal tail region, suggesting that these N-terminal residues may function to recruit TSH2B-specific nucleosome remodellers and histone chaperones, which could be required for histone exchange during spermatogenesis. Intriguingly, another testis-specific histone variant, H3T, is not efficiently incorporated into nucleosomes by the conventional histone chaperone Nap1, but can be assembled by the histone chaperone Nap2 (Tachiwana *et al.*, 2008 ▶). The expression of Nap2 is threefold higher in testis than in other tissues (Hu *et al.*, 1996 ▶). A TSH2B-specific chaperone that possibly binds to its N-terminal region may exist and function together with Nap2 during the chromatin transition stages in human spermatogenesis.

## Supplementary Material

PDB reference: TSH2B nucleosome, 3wkj

## Figures and Tables

**Table 1:** Data-collection and refinement statistics for the TSH2B nucleosome Values in parentheses are for the highest resolution shell.

Data collection	
Space group	*P*2_1_2_1_2_1_
Unit-cell parameters (Å)	*a* = 107.0370, *b* = 109.9260, *c* = 182.9610
Resolution (Å)	50.0–2.80 (2.90–2.80)
No. of reflections	1075793
No. of unique reflections	54290
Completeness (%)	98.9 (90.9)
*R*_merge_ † (%)	7.9 (50.4)
〈*I*/σ(*I*)〉	7.0 (4.3)
Multiplicity	7.3 (6.6)
Refinement	
Resolution (Å)	47.24–2.80
*R*_work_ ‡/*R*_free_ (%)	23.5/28.7
*B* factors (Å^2^)	
Protein	46.9
DNA	111.4
R.m.s. deviations	
Bond lengths (Å)	0.007
Bond angles (°)	1.16
Ramachandran favoured (%)	96.3
Ramachandran outliers (%)	0.0
PDB code	3wkj

† *R*_merge_ = 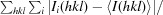 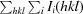.

‡ *R*_work_ = 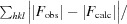 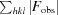. *R*_free_ was calculated with 5% of the data excluded from the refinement.
